# 10-mer and 9-mer WALK Peptides with Both Antibacterial and Anti-Inflammatory Activities

**DOI:** 10.3390/antibiotics11111588

**Published:** 2022-11-10

**Authors:** Su-Jin Kim, Tae-Bong Kang, Dong-Hyuk Kim, Minho Keum, Sung-Hee Lee, Ji-Hun Kim, Sang-Hyuck Lee, Jihoon Kim, Hyuk-Jung Kweon, Jae-Won Park, Beom-Jun Kim, Hyung-Sik Won

**Affiliations:** 1 BK21 Project Team, Department of Applied Life Science, Graduate School, Konkuk University, Chungju 27478, Korea; 2 Department of Biotechnology, Research Institute (RIBHS), College of Biomedical and Health Science, Konkuk University, Chungju 27478, Korea; 3 College of Pharmacy, Chungbuk National University, Cheongju 28160, Korea; 4 Department of Family Medicine, School of Medicine, Konkuk University, Chungju 27478, Korea; 5 Research Institute, EYESEL Co., Ltd., Yongin 16950, Korea

**Keywords:** antimicrobial peptides, host defense peptides, de novo design, WALK peptides, antibacterial activity, anti-inflammatory activity

## Abstract

Natural antimicrobial peptides (AMPs) are multifunctional host defense peptides (HDPs) that are valuable for various therapeutic applications. In particular, natural and artificial AMPs with dual antibacterial immunomodulatory functions emerged as promising candidates for the development of therapeutic agents to treat infectious inflammation. In an effort to develop useful AMP variants with short lengths and simple amino acid composition, we devised a de novo design strategy to generate a series of model peptide isomer sequences, named WALK peptides, i.e., tryptophan (W)-containing amphipathic-helical (A) leucine (L)/lysine (K) peptides. Here, we generated two groups of WALK peptide isomers: W_2_L_4_K_4_ (WALK244.01~WALK244.10) and W_2_L_4_K_3_ (WALK243.01~WALK243.09). Most showed apparent antibacterial activities against both Gram-positive and Gram-negative bacteria at a concentration of approximately 4 μg/mL along with varied hemolytic activities against human red blood cells. In addition, some exhibited significant anti-inflammatory activities without any significant cytotoxicity in macrophages. Collectively, these results suggest that the two selected peptides, WALK244.04 and WALK243.04, showed promise for the development of antibacterial and anti-inflammatory agents.

## 1. Introduction

As global public health faces a looming crisis due to the emergence, evolution, and worldwide spread of antibiotic-resistant bacteria, alternative antibiotic agents with different mechanisms are urgently needed. At this conjuncture, antimicrobial peptides (AMPs), which selectively disrupt the bacterial cell’s membrane, captured attention as highly promising agents for combating drug-resistant bacteria [1,2]. In practice, the administration of AMPs, together with an adjunctive antibiotic, was the sole cure for some cases of infectious diseases, such as sepsis and skin infections [1]. Natural AMPs, which are characterized as genetically encoded endogenous antibiotic peptides, have been identified in all domains of life and protect the host from invading pathogens [3,4,5]. Accordingly, many AMPs and their variants have been considered potential sources of novel antibiotic agents for clinical and industrial applications [6,7,8,9]. However, the therapeutic potential of AMPs associated with human health and diseases is not limited solely to their antimicrobial action, as some AMPs possess pleiotropic activities, including anti-cancer, anti-diabetic, anti-inflammatory, wound-healing, and anti-COVID-19 effects [4,6,10,11]. In particular, as immunomodulatory activities against infectious microbes are the complementary functions of many natural AMPs, they are recognized as host defense peptides (HDPs) that are components of the innate immunity system [12,13]. Consequently, AMPs/HDPs with antimicrobial and immunomodulatory dual functions show promise in the development of novel therapeutic agents for treating infectious inflammation [14,15,16]. The desirable features of AMPs/HDPs as anti-infectious agents include a broad-spectrum bactericidal activity, rapid microbial killing, synergistic effects with conventional antibiotics, and a low propensity for resistance development [17,18]. Immunomodulatory AMPs/HDPs that target the host immune system instead of the pathogen can also minimize the risk of pathogen resistance to these molecules [16]. Therefore, many peptide molecules that have been modified or designed based on the structural features of natural AMPs/HDPs are used in clinical and commercial development [3,8,19]. For instance, omiganan (ILRWPWWPWRRK-NH_2_), a dodecapeptide variant derived from indolicidin, is currently undergoing clinical trials as an antibacterial and anti-inflammatory agent [20,21], which was used as a positive control in this study. However, the poor pharmacokinetic properties and high manufacturing costs of AMPs/HDPs are major obstacles to their pharmaceutical and industrial applications [9,22]. To address these limitations, we attempted to develop novel AMP molecules with short lengths and simple amino acid composition [23,24,25], which would be highly beneficial for reducing production costs and facilitating optimized pharmaceutical production [13,15,26]. Although naturally occurring AMPs/HDPs are extremely diverse in sequence and structure, cationic, amphipathic α-helical peptides represent a particularly abundant, widespread, and well-characterized group [19,27,28]. Their amphipathic helical conformation, with formations that are typically induced upon membrane interaction, is critical for killing bacteria via membrane permeation, while positive charges are required for the discrimination of the anionic surface of bacterial membranes from zwitterionic eukaryotic membranes. Based on these structural insights, we previously developed a simple strategy to design short model peptides, namely WALK peptides, i.e., tryptophan (W)-containing amphipathic-helical leucine (L)/lysine (K) peptides, which mimic the cationic, amphipathic α-helical group of AMPs/HDPs using only three types of amino acids [23,24,25]. In this design, leucines and lysines form the fundamental scaffold of amphipathic α-helix, whereas tryptophan, which generally enhances the membrane-binding ability of the peptides, is located at the leucine/lysine interface to stabilize the helical conformation and balance the amphipathic property. Since we attempted to generate less than 12-mer AMPs considering that natural AMPs/HDPs typically consist of 12–50 amino acid residues [26], we created WALK155 undecapeptide isomers with the L_5_K_5_W formula [23]. Some of the WALK155 peptides showed strong antibacterial activity with little hemolytic activity. Among them, the WALK155.03 (LKWLKKLLKKL-NH_2_) peptide exhibited a potent anti-inflammatory activity without cytotoxicity [29]. In the present study, to develop more improved (i.e., shorter and more active) candidates than WALK155.03, we designed and screened WALK decapeptides and nonapeptides for antibacterial and anti-inflammatory (ABAI) dual functions.

## 2. Results

### 2.1. WALK Peptide Design and Conformational Validation

**Figure 1:**
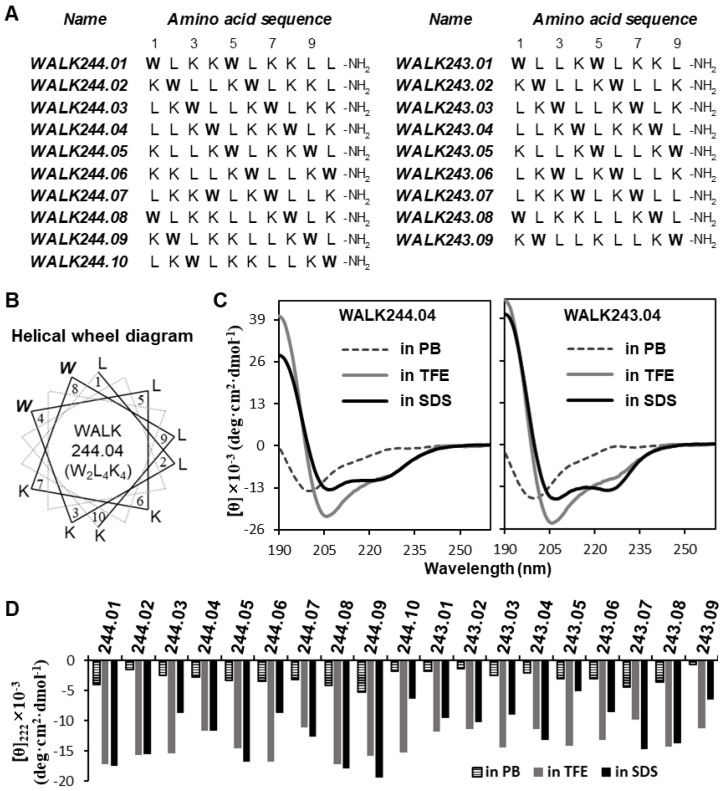
Sequence design and conformational validation of WALK peptides. (**A**) Amino acid sequences of individual WALK244 and WALK243 peptides. Tryptophan (W) residues are indicated in bold. (**B**) An example of a helical wheel diagram illustrated for the WALK244.04 peptide. The diagrams for other peptides are provided in Supplementary Figure S1. (**C**) Examples of far-UV CD spectra demonstrated for WALK244.04 and WALK243.04 peptides. The spectra of the other peptides are represented in Supplementary Figure S2. Individual peptide samples (100 μM) were dissolved in 10 mM sodium phosphate buffer alone (dashed line; PB), PB containing 50% (*v*/*v*) trifluoroethanol (gray line; TFE), and PB containing 10 mM sodium dodecyl sulfate (black solid line; SDS). (**D**) Comparison of CD spectroscopic parameters. Mean residue molar ellipticities of each WALK peptide at 222 nm ([θ]_222_) in different solvents are summarized: PB (scratched bar), TFE (gray bar), and SDS (black bar).

Figure 1A summarizes the amino acid sequences of the amphipathic helical model peptides tested, which comprised only three types of amino acids: two tryptophan, four leucine, and four or three lysine residues. The two groups, termed WALK244 and WALK243, comprised ten decapeptide isomers with the W_2_L_4_K_4_ formula and nine nonapeptide isomers with the W_2_L_4_K_3_ formula, respectively. According to our WALK design scheme, a helical-wheel diagram was used to devise perfectly amphipathic peptide sequences when folded into α-helical structures, and it was used to locate the two tryptophans at the critical amphipathic interface between the side with the ending lysyl and that with the starting leucyl (Figure 1B and Supplementary Figure S1). The decimal digits of individual WALK serial numbers denote the residue numbers of the first tryptophan residue neighboring the ending lysyl side in the helical-wheel projection. All peptides were designed to be amidated at their C-termini to remove the C-terminal’s negative charge at a neutral pH.

As a helical conformation is a fundamental requirement for our design concept, the helical formation of chemically synthesized WALK peptides was ascertained by CD spectroscopy. The far-UV CD spectra of WALK244.04 and WALK243.04 as representative examples are shown in Figure 1C, and the spectra for the other peptides are presented in Supplementary Figure S1. In addition, the mean residue molar ellipticity ([θ]) value at 222 nm, which is highly sensitive to the α-helix content, is summarized in Figure 1D for individual WALK peptides. As typically observed for amphipathic α-helical AMPs, all our WALK peptides also showed common conformational behavior depending on the solvents. A strong negative band observed around 200 nm, a characteristic of predominantly disordered conformation in an aqueous buffer was compensated by the signal intensification at 208 and 222 nm, which is indicative of increased α-helix contents in environments containing 50% TFE or 10 mM SDS micelles. These results suggest that WALK peptides are likely to adopt α-helix-dominated conformations upon interactions with bacterial biomembranes, and the antimicrobial action of the peptides could be anticipated via their amphipathic helical properties, as assumed in the design step.

### 2.2. Evaluation of Antibacterial and Hemolytic Activities

Table 1 summarizes the minimal inhibitory concentration (MIC) values of WALK peptides and two conventional antibiotics, ampicillin and kanamycin, against two Gram-positive and two Gram-negative bacterial strains. All WALK244 and WALK243 peptides showed antibacterial activities against all strains tested, with MIC values ranging from 1 to 32 μg/mL, some of which were comparable to the MIC values of the β-lactam antibiotic ampicillin and protein synthesis inhibitor kanamycin. The normalized geometric mean of MICs (GM) values of WALK peptides against the four strains ranged from 1.7 to 11.3 μg/mL, yielding an average GM value of 4.1 ± 2.6 μg/mL, which is equivalent to 3.0 ± 1.9 μM of WALK244 and 3.3 ± 2.6 μM of WALK243 peptide isomers. Among the 19 WALK peptides, the most potent activity (i.e., the lowest GM value) was observed with WALK244.01 and WALK244.09, whereas WALK244.03 showed the highest GM value (i.e., the weakest activity). Overall, the antibacterial activities observed for all WALK peptides confirmed that our de novo peptide design strategy was highly effective in generating a broad range of antibacterial molecules that are comparable in potency to conventional antibiotics.

**Figure 2:**
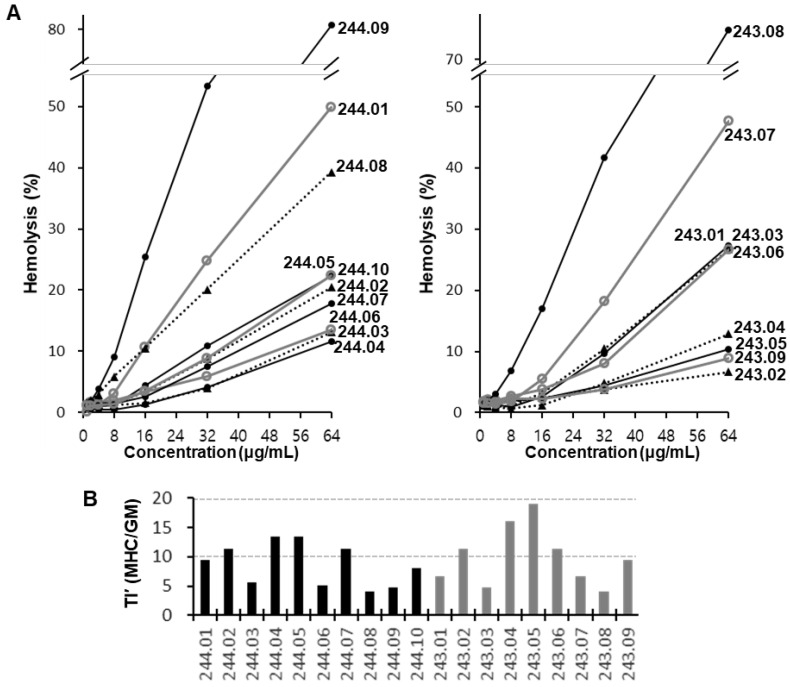
Hemolytic activity of WALK peptides. (**A**) Percentage hemolysis of human red blood cells treated with the serially diluted WALK244 and WALK243 peptides. (**B**) Pseudo-therapeutic index (TI’) of WALK peptides deduced from minimal hemolytic concentration (MHC) and antibacterial GM (geometric mean of minimal inhibitory concentrations) values.

To evaluate the potential use of our WALK peptides as therapeutic candidates, their hemolytic activities against human erythrocytes were examined (Figure 2A). As previously observed for WALK155 undecapeptide isomers [23], the present WALK244 and WALK243 isomers also showed remarkable variations in hemolytic activity. Based on the percentage hemolysis values at a concentration of 64 μg/mL, WALK244.09 and WALK243.02 peptides appeared to be the most (80.7%) and the least (6.7%) hemolytic, respectively. In order to estimate the pseudo-therapeutic index (TI’) values of individual peptides (Figure 2B), we determined the minimal hemolytic concentration (MHC) of each peptide (Table 1) at a minimal concentration showing 5% or more hemolysis in Figure 2A. Although it is distinct from the typical therapeutic index for chemotherapy, TI’ could be useful for the relative comparison of safety between the peptides via a simple assessment of the balance between the hemolytic dose represented by the MHC value and the antibacterial dose represented by the GM value (TI’ = MHC/GM) [24,30,31]. Based on this, WALK244.02, 244.04, 244.05, 244.07, 243.02, 243.04, 243.05, and 243.06 peptides could be categorized into a relatively more favorable group for the development of therapeutic antibacterial peptide agents.

### 2.3. Screening for Immunomodulatory Potentials

**Figure 3:**
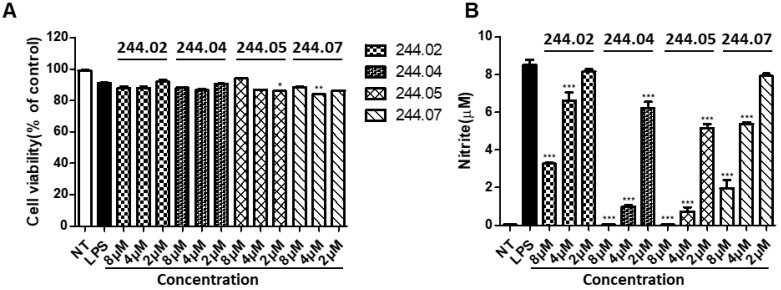
Initial screening of selected WALK244 peptides for immunomodulatory potential in J774A.1 cells. All bar graphs represent the mean ± SEM of three independent experiments (* *p* < 0.05, ** *p* < 0.01, and *** *p* < 0.001, significantly different from the control group treated with LPS alone). NT, non-treated; LPS, lipopolysaccharides. (**A**) Cytotoxicity was assessed using the MTT assay. The cells were treated with lipopolysaccharides (LPS, 100 ng/mL) or peptides (8–2 μM, two-fold serial dilutions) for 16 h, followed by estimating the cell viability as the percentage of surviving cells compared to that of the control cells. (**B**) Anti-inflammatory potential deduced by NO assay. Inhibitory effects of pretreated individual peptides on NO release in the cells stimulated by LPS (100 ng/mL) for 16 h were evaluated by measuring the nitrite concentrations in the culture supernatants.

To develop useful ABAI peptides, the anti-inflammatory activities of our WALK peptides were examined after serial dilutions to obtain concentrations (8, 4, and 2 μM) that were around their antibacterial GM values. WALK244.02, 244.04, 244.05, and 244.07, which showed relatively higher (>10) TI’ values (Figure 2B), were selected in the WALK244 group for the initial screening of the immunomodulatory potential in the macrophage J774A.1 cell line. All four peptides showed no significant cytotoxicity in the 3-[4,5-dimethylthiazole-2-yl]-2,5-diphenyltetrazoliumbromide (MTT) assay (Figure 3A), whereas a dose-dependent inhibitory effect against NO production was observed in lipopolysaccharides (LPS)-stimulated J774A.1 cells (Figure 3B). In particular, WALK244.04 and WALK244.05 peptides appeared to be the most potent, showing near-complete NO inhibition at 8 μM. 

**Figure 4:**
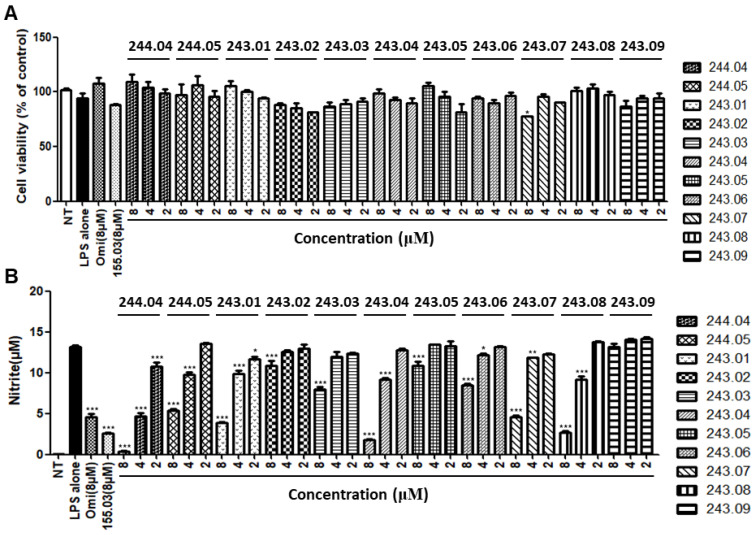
Screening of two selected WALK244 and all WALK243 peptides for immunomodulatory potential in Raw264.7 cells. Known anti-inflammatory peptides, WALK155.03 (8 μM) and omiganan (Omi, 8 μM), were compared as positive controls. All bar graphs represent the mean ± SEM of three independent experiments (* *p* < 0.05, ** *p* < 0.01, and *** *p* < 0.001, significantly different from the control group treated with LPS alone). Cytotoxicity (**A**) was assessed as cell viability, while the NO inhibitory effect (**B**) was deduced by measuring nitrite concentrations in the culture supernatants, as described in Figure 3.

Hence, confirmatory experiments for the NO inhibitory activity of these two peptides were conducted in other macrophage RAW264.7 cells (Figure 4). In this step, the known anti-inflammatory peptide omiganan and previously developed undecapeptide WALK155.03 were used as positive controls; all WALK243 peptide isomers were also examined for comparison. The results suggested that WALK244.04 had the most potent NO inhibitory activity, which was even stronger than that of omiganan and WALK155.03. In the case of shorter WALK243 nonapeptides, members with high TI’ (WALK243.02, 243.04, 243.05, and 243.06; Figure 2B) also did not demonstrate significant cytotoxicity at the concentrations tested (Figure 4A). Among them, WALK243.04 (8 μM) showed superior NO inhibition compared to other positive controls. Collectively, our screening for examination of immunomodulatory potential by NO assay suggested that the two peptides, WALK244.04 in the decapeptide group and WALK243.04 in the nonapeptide group, were the most promising members likely with dual ABAI activities and little toxicity (hemolytic activity and cytotoxicity). Therefore, these two peptides were selected for subsequent studies of anti-inflammatory activities.

### 2.4. Validation of Anti-Inflammatory Activities of Selected WALK Peptides

**Figure 5:**
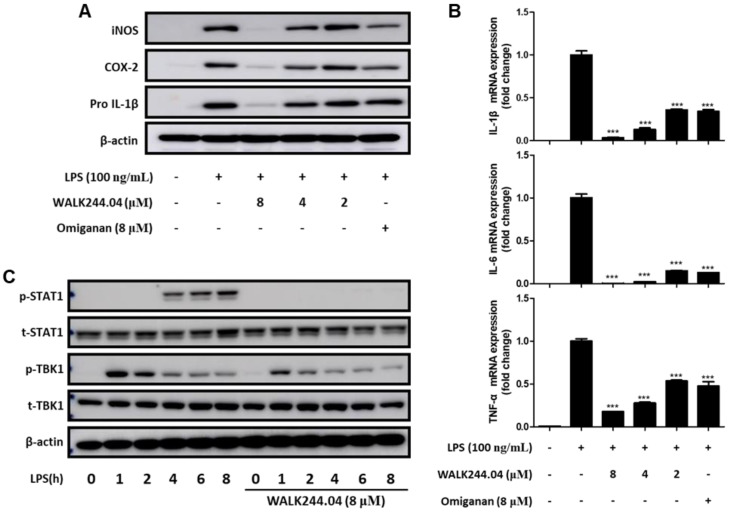
Anti-inflammatory effects of WALK244.04 peptide in Raw264.7 cells. The cells were pretreated with the peptide at indicated concentrations for 5 min, followed by stimulation with LPS (100 ng/mL) to induce inflammatory responses. (**A**) iNOS, COX-2, and pro-IL-1β expression levels were estimated by Western blot analysis after 16 h of LPS treatment. (**B**) The mRNA expression of IL-1β, IL-6, and TNF-α was determined by quantitative real-time PCR upon 3 h of stimulation with LPS. All bar graphs represent the mean ± SEM of three independent experiments (*** *p* < 0.001, significantly different from the control group treated with LPS alone). (**C**) The phosphorylation of STAT1 and TBK1 was examined by Western blot analysis at different time points after the LPS treatment.

The dose-dependent effects of WALK244.04 on the production of pro-inflammatory mediators in LPS-stimulated RAW264.7 cells were evaluated by immunoblotting and real-time qPCR. The remarkable inhibition of NO production observed by the peptide at 8 μM (Figure 4B) was validated by confirming the substantial downregulation of cognate iNOS gene expression (Figure 5A). Likewise, the downregulation of the expression of other pro-inflammatory mediators, COX-2 and pro-IL-1β, was also evident in the immunoblot analysis (Figure 5A). Real-time qPCR results also indicated that the peptide effectively suppressed the mRNA expression of three major pro-inflammatory cytokines: IL-1β, IL-6, and TNF-α (Figure 5B). In addition, consistent with the results of the NO assay (Figure 4B), the anti-inflammatory effects of WALK244.04 at a concentration of 8 μM appeared to be more potent than those of omiganan at the same concentration.

Finally, as our previous study on WALK155.03 revealed that the anti-inflammatory action of the peptide was attributed to the inhibition of the TRIF-dependent signaling pathway upon TLR4 stimulation [29], we investigated whether WALK244.04 could also attenuate the TRIF-dependent pathway involved in TLR4-mediated pro-inflammatory signaling (Figure 5C). TRIF-dependent signaling, which is triggered by association with internalized TLR4 upon stimulation by LPS, is known to be mediated by the phosphorylation of the downstream kinase TANK binding kinase-1 (TBK-1) to produce type-I interferons [32]. These interferons induce the expression of downstream target genes via the phosphorylation of the transcription factor signal transducer and the activator of transcription-1 (STAT-1) [33]. Therefore, we confirmed that the WALK244.04 peptide attenuated TBK-1 phosphorylation at an early stage (after 1 h of LPS treatment), which resulted in the strong inhibition of STAT-1 phosphorylation at a later stage (after 4 h of LPS treatment). These results indicated the likely involvement of TRIF signaling inhibition in mediating the anti-inflammatory effect of the peptide.

**Figure 6:**
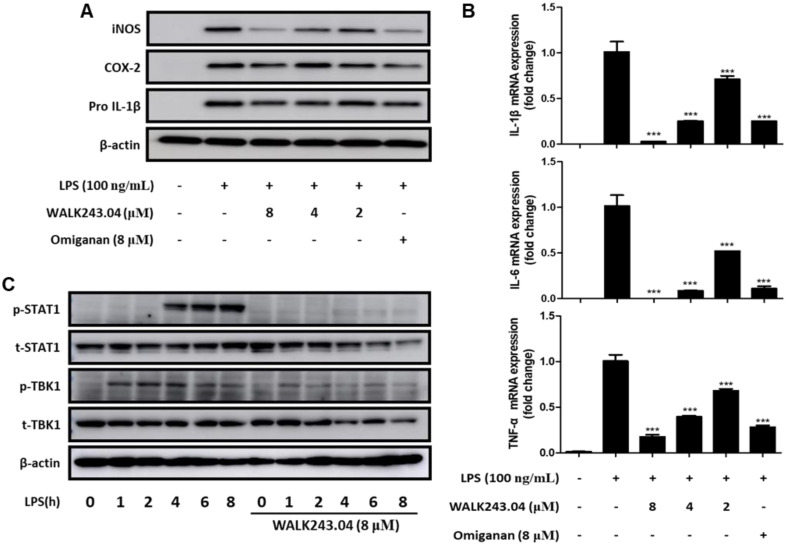
Anti-inflammatory effects of WALK243.04 peptide in Raw264.7 cells. The cells were pretreated with the peptide at indicated concentrations for 5 min, followed by stimulation with LPS (100 ng/mL) to induce inflammatory responses. (**A**) iNOS, COX-2, and pro-IL-1β expression levels were estimated by Western blot analysis after 16 h of LPS treatment. (**B**) The mRNA expression of IL-1β, IL-6, and TNF-α was determined by quantitative real-time PCR after 3 h of stimulation with LPS. All bar graphs represent the mean ± SEM of three independent experiments (*** *p* < 0.001, significantly different from the control group treated with LPS alone). (**C**) The phosphorylation of STAT1 and TBK1 was analyzed by Western blot analysis at different time points after LPS treatment.

Most anti-inflammatory properties observed for the decapeptide WALK244.04 were also relevant to the action of the WALK243.04 nonapeptide (Figure 6). However, the anti-inflammatory potency of WALK243.04 was lower than that of WALK244.04, as the inhibitory effects of WALK243.04 on the pro-inflammatory mediators including iNOS, COX-2, and pro-IL-1β (Figure 6A) were estimated to be less potent or weaker than those of WALK244.04 (Figure 5A). Nonetheless, WALK243.04 may be a promising anti-inflammatory peptide, as its anti-inflammatory activity was comparable to or higher than that of the positive control omiganan.

## 3. Discussion

For novel AMP/HDP development, reducing the peptide size is required to facilitate the future optimization of pharmaceutical and pharmacokinetic properties as well as to reduce production costs [13,15,26]. The present study attempted to develop short AMPs with ten or less amino acid residues while adopting amphipathic helical scaffolds. General approaches for generating new AMP/HDP variants include the sequence modification of natural AMPs/HDPs, screening combinatorial libraries, template-assisted designs, and a minimalist approach to de novo designs [9,28]. Among them, our WALK peptides were generated via de novo designs by considering the structural properties of the cationic and amphipathic α-helical groups of AMPs. Although helical stabilization in membrane environments is a prerequisite for the antibacterial action of these peptides, an elaborate design of the AMP sequences for potent antibacterial activity with little hemolytic activity is highly challenging because many other structural parameters other than helicity are associated with one another, including the peptide’s length, mean residue hydrophobicity, amphipathicity, hydrophobic moment, net positive charge, and polar angle in a sophisticated manner to modulate and balance the antibacterial activity and selectivity [34]. Using our de novo design strategy, a small group of peptide isomers with potent antibacterial activity and varied hemolytic activity are generated using only three types of amino acids. Among the designed group members, the best peptide with the most desirable activity and selectivity is selected via activity screening. In this manner, the present study generated two groups of new WALK peptide isomers, each with ten or nine amino acid residues. As most of them showed potent antibacterial activities against both Gram-positive and Gram-negative bacteria, the results validated again that our WALK strategy can serve as a highly effective approach for the development of novel AMP molecules.

In WALK peptides, leucine and lysine residues provide a fundamental framework for the amphipathic helical structure, whereas tryptophan residues incorporated at the critical amphipathic interface play a key role in the bioactivity of peptides by stabilizing the amphipathic helical conformation and reinforcing the membrane’s affinity of the peptides [35,36,37]. In this regard, most WALK peptides that we previously studied had a single tryptophan residue, as the hemolytic activity, as well as the antibacterial activity, was also strengthened when two tryptophan residues were incorporated [24,36]. Meanwhile, both antimicrobial and hemolytic activities diminished when the length of the single-tryptophan WALK peptides was reduced to under 11 residues [24,25]. Therefore, we surmise that by incorporating the two contrary factors (i.e., using double tryptophans and reducing the peptide length) together in the design strategy, the present study could be successful in finding promising WALK peptides with balanced activity (i.e., potent antimicrobial activity with little hemolytic activity), even with their challenging (shorter than 11 residues) length.

Although many amphipathic helical AMPs demonstrate anti-inflammatory activities, generalized or conserved structural determinants or the exact modes of action for this immunomodulatory activity have not yet been clearly elucidated due to the high sequence diversity of AMPs. The inflammatory response to endo- and exo-stimuli, which is crucial for host survival, is responsible for many chronic inflammatory diseases [38]. Activated macrophages play a critical role in infectious inflammation, and LPS, a representative macrophage stimulator is most widely used to induce the rapid activation of innate immune responses in host cells [39,40]. LPS-induced macrophage responses are typically considered as anti-inflammatory targets, thereby prompting an active search for substances as potential anti-inflammatory agents that effectively inhibit the pro-inflammatory mediators produced by macrophages. In this context, we tested the anti-inflammatory potential of WALK peptides in an LPS-stimulated macrophage model. Although the LPS-binding ability required for the antibacterial action of some AMPs has been simply regarded to be associated with their LPS-neutralizing anti-inflammatory activity mediated via scavenging LPS [41], our previous investigation using WALK155.03 demonstrated that the specific inhibition of the TRIF-dependent pathway of LPS-stimulated TLR4 signaling was an alternative, major route used to exert anti-inflammatory activities [29]. The present study also confirmed that TRIF-dependent signaling can be suppressed by WALK244.04 and WALK243.04 peptides. Therefore, given their structural resemblance, we assume that the function of the present WALK peptides would be similar to that of WALK155.03 with respect to the anti-inflammatory effect, although the detailed mechanism remains to be further validated.

In summary, based on the WALK design strategy, ten decapeptide and nine nonapeptide isomers were screened for useful ABAI duality. Among them, the WALK244.04 decapeptide was found to have the most desirable bioactivity. It showed MIC values of approximately 4 μg/mL against both Gram-positive and Gram-negative bacterial strains, whereas no significant hemolytic activity was observed at concentrations under 64 μg/mL. In addition, its anti-inflammatory activity without cytotoxicity in macrophages was superior to that of the known dodecapeptide omiganan and the previously developed WALK155.03 undecapeptide. Alternatively, the WAL243.04 peptide also showed comparable bioactivity and safety to WALK244.04. Although the anti-inflammatory activity of WAL243.03 appeared to be somewhat lower than that of WALK244.04, WAL243.04 is one amino acid residue shorter than WALK244.04, which is beneficial for commercial production. Therefore, we suggest that WALK244.04 and WALK243.04 are the most promising ABAI peptide molecules for the future therapeutic development of pharmaceutical or cosmeceutical agents against infectious inflammation.

## 4. Materials and Methods

### 4.1. Materials and Peptide Preparation

The murine macrophage J774A.1 and RAW264.7 cell lines were purchased from the Korean Cell Line Bank (Seoul, Korea) and maintained in RPMI medium (Thermo Fisher Scientific, Seoul, Korea) supplemented with 10% fetal bovine serum and 1% penicillin-streptomycin at 37 °C in a humidified atmosphere containing 5% CO_2_. Monoclonal antibodies for immunoblotting assays were purchased from Cell Signaling Technology Inc. (Danvers, MA, USA) and Santa Cruz Biotechnology Inc. (Dallas, TX, USA). The antibodies are listed as follows: iNOS (CST 13120S), COX-2 (CST 12282S), IRF3 (CST 4302S), TBK1/NAK (CST 51872S), STAT1 (CST 9172S), Phospho-TBK1/NAK (CST 5483S), Phospho-Stat1 (CST 9167S), and β-actin (SCB sc-4778). Chemically synthesized WALK peptides and omiganan were purchased as dry powders from the peptide manufacturing company AnyGen (Kwangju, Korea). For experiments, each peptide powder was dissolved in its designated solvent, followed by a spectrophotometric measurement of concentration performed using the known value of molar absorptivity for tryptophan at 280 nm, i.e., 5500 for single tryptophan (WALK155.03 peptide), 11,000 for two tryptophan (WALK244 and WALK243 peptides), and 22,000 M^−1^·cm^−1^ for four tryptophan residues (omiganan).

### 4.2. Circular Dichroism (CD) Spectroscopy

Far-UV CD experiments were performed with individual peptides at 0.1 mM concentration dissolved in the following three different solvents at pH 6.7: 10 mM sodium phosphate buffer (PB) and PB containing 50% (*v/v*) trifluoroethanol (TFE) or 10 mM sodium dodecyl sulfate (SDS). All CD spectra were recorded on a JASCO J-715 spectropolarimeter at 20 °C using a cell with a path length of 0.1 cm, a bandwidth of 1 nm, and a step resolution of 0.2 nm. For each spectrum, three individual scans taken from 260 to 190 nm were summed and averaged, followed by subtraction of the solvent CD signal. Finally, the CD intensity recorded in units of mdeg at each wavelength was normalized to the mean residue molar ellipticity ([θ]) in deg∙cm^2^·dmol^−1^ [35].

### 4.3. Antimicrobial Assay

The antimicrobial activity of each peptide was assessed against four strains, including two Gram-positive (*Bacillus subtilis* ATCC 6633 and *Staphylococcus aureus* ATCC 6538p) and two Gram-negative bacteria (*Escherichia coli* ATCC 25922 and *Shigella dysentariae* ATCC 9752) cultured in Luria-Bertani broth media. Antibacterial susceptibility was determined as the MIC value, which was measured using the standard broth microdilution method as described previously [24,36]. Briefly, MIC was defined as the lowest peptide concentration that completely inhibited cell growth in the presence of various concentrations (1–64 μg/mL, two-fold serial dilutions) of peptides. Two conventional antibiotics, ampicillin and kanamycin, were used as controls to confirm antibiotic susceptibility. The tests were performed in triplicate, and the MIC values that were reproduced twice or thrice in the three independent measurements were employed to calculate the GM value.

### 4.4. Hemolytic Assay

Hemolysis experiments were conducted as previously described [24,36]. Briefly, suspensions of human red blood cells (10% *v*/*v* in PBS) were treated for 1 h with various concentrations (1–64 μg/mL, two-fold serial dilutions) of peptides, followed by measurement of the absorbance of the supernatant at 550 nm. The relative attenuation was determined as the percentage of hemolysis compared to that of the blood suspension treated with 0.2% Triton X-100. The tests were performed in triplicate, and the average values of three independent measurements were recorded. The lowest peptide concentration that showed 5% or more hemolysis [30,31] was determined as the minimal hemolytic concentration (MHC), which was used to calculate the TI’ value (TI’ = MHC/GM).

### 4.5. Cell Viability Test

Macrophage cells seeded in 96-well cell culture plates at a density of 3 × 10^4^ cells/well were incubated for 24 h, followed by treatment with LPS or individual WALK peptides and subsequent incubation for 16 h. Cell viability was then assessed by the conventional MTT assay. Briefly, the medium in each well was replaced with 100 μL of RPMI medium containing MTT (500 μg/mL). After 2 h of incubation, the medium was discarded and the insoluble formazan in each well was dissolved in 100 μL of DMSO with shaking. The absorbance of the solution at 550 nm was measured using a spectrophotometric microplate reader to determine the percentage of cell viability relative to that of control cells.

### 4.6. Estimation of Nitric Oxide (NO) Production

The macrophage cells were seeded (3 × 10^4^ cells/well) and incubated in 96-well plates for 24 h, followed by pretreatments with the WALK peptides at designated concentrations for 5 min and subsequent stimulation with 100 ng/mL of LPS for 16 h. The nitrite concentration in the culture medium was determined using the Griess reaction assay. Briefly, the culture supernatant (100 μL) was mixed with an equal volume of Griess reagent (1% sulfanilamide, 0.1% *N*-1-naphthyl ethylenediamine) and incubated for 5 min. The absorbance of the resulting chromophoric azo-derivative molecules was measured at 550 nm using a microplate reader with a fresh culture medium as a blank. The amount of nitrite in each sample was determined using a standard curve generated using a range of dilutions of sodium nitrite solutions.

### 4.7. Immunoblot Analysis

The cells were lysed using a RIPA buffer (iNtRON) containing protease and phosphatase inhibitor cocktails. The cell lysates were resolved using 10% SDS-PAGE and transferred to nitrocellulose membranes, which were blocked for 1 h at room temperature (approximately 23 °C) using phosphate-buffered saline with 0.01% Tween-20 (PBST, Merck Korea, Seoul, Korea) containing 10% fat-free dried milk. The blocked membranes were then incubated overnight with primary antibodies at 4 °C, followed by further incubation with horseradish peroxidase-conjugated secondary antibodies for 1 h at room temperature. The membranes were developed using the enhanced chemiluminescence (ECL) detection kit (Advansta, San Jose, CA, USA) and visualized using a luminescent image analyzer (Amersham imager 680, Cytiva Korea, Incheon, Korea).

### 4.8. Quantitative Real-Time PCR

The cells were stimulated with LPS (100 ng/mL) for 3 h in the presence or absence of peptide samples, and the total RNA was extracted using a HiGene™ Total RNA Prep Kit (BIOFACT, Daejeon, Korea). cDNA was synthesized from 500 ng of total RNA using a SuperiorScript III cDNA synthesis kit (Enzynomics, Daejeon, Korea). Real-time PCR was then performed on a Lightcycler 96 instrument (Roche Diagnostics Korea, Seoul, Korea), with a Dyne qPCR 2X PreMIX (Dyne Bio, Seongnam, Korea) and the following gene-specific primers (sense and antisense primer sequences, respectively): 5′-TGGACCTTCCAGGATGAGGACA-3′ and 5′-GTTCATCTCGGAGCCTGTAGTG-3′ for IL-1β, 5′-TACCACTTCACAAGTCGGAGGC-3′ and 5′-CTGCAAGTGCATCATCGTTGTTC-3′ for IL-6, and 5′-GGTGCCTATGTCTCAGCCTCTT-3′ and 5′-GCCATAGAACTGATGAGAGGGAG-3′ for TNF-α. The resulting 2^−ΔΔCt^ value for each group was used to calculate the relative expression ratio of the detected mRNA.

### 4.9. Statistics

Statistical analyses were performed using GraphPad Prism 5 software (www.graphpad.com, accessed on 6 October 2022). Data were represented as mean ± SEM for three independent experiments. Statistical differences were assessed using one-way ANOVA followed by Dunnett’s multiple comparison test. In all comparisons, values of * *p* < 0.05, ** *p* < 0.01, and *** *p* < 0.001 were considered to indicate significant differences.

## Figures and Tables

**Table 1:** Antibacterial and hemolytic activities of WALK244 and WALK243 peptide isomers.

WALK Serial Number	MIC ^1^ (μg/mL)					MHC ^3^ (μg/mL)
	Gram-Positive Bacteria		Gram-Negative Bacteria		GM ^2^ (μg/mL)	
	*B. subtilis*	*S. aureus*	*E. coli*	*S. dysentariae*		
244.01	≤1	2	2	2	≤1.7	16
244.02	2	2	2	8	2.8	32
244.03	8	8	8	32	11.3	64
244.04	4	4	4	8	4.8	64
244.05	2	2	2	4	2.4	32
244.06	2	3	16	16	6.3	32
244.07	2	2	2	8	2.8	32
244.08	2	≤1	2	4	≤2.0	8
244.09	≤1	2	2	2	≤1.7	8
244.10	2	2	8	8	4.0	32
243.01	4	4	4	8	4.8	32
243.02	4	4	4	16	5.7	64
243.03	4	4	8	16	6.7	32
243.04	4	4	4	4	4.0	64
243.05	2	2	2	16	3.4	64
243.06	2	2	2	8	2.8	32
243.07	2	2	2	4	2.4	16
243.08	2	2	2	2	2.0	8
243.09	4	4	8	16	6.7	64
Ampicillin	≤1	≤1	≤1	≤1	≤1	-
Kanamycin	4	4	4	8	4.8	-

^1^ MIC, minimal inhibitory concentration; ^2^ GM, geometric mean of MICs; ^3^ MHC, minimal hemolytic concentration.

## Data Availability

Not applicable.

## Supplementary Materials

The following supporting information can be downloaded at: https://www.mdpi.com/article/10.3390/antibiotics11111588/s1, Figure S1: Helical wheel diagrams for designing WALK244 and WALK243 peptide sequences; Figure S2: Far-UV CD spectra of WALK244 and WALK243 peptides.

## Abbreviations

ABAI	antibacterial and anti-inflammatory
AMP	antimicrobial peptide
CD	circular dichroism
CL	enhanced chemiluminescence
GM	geometric mean of minimal inhibitory concentrations
HDP	host defense peptide
LPS	lipopolysaccharides
MHC	minimal hemolytic concentration
MIC	minimal inhibitory concentration
MTT	3-[4,5-dimethylthiazole-2-yl]-2,5-diphenyltetrazoliumbromide
PB	phosphate buffer
SDS	sodium dodecyl sulfate
STAT	signal transducer and activator of transcription
TBK	kinase TANK binding kinase
TFE	trifluoroethanol
TI’	pseudo-therapeutic index
WALK	tryptophan-containing amphipathic-helical leucine/lysine
