# Supplementary material for: 10-mer and 9-mer WALK Peptides with Both Antibacterial and Anti-Inflammatory Activities
Source: Antibiotics (Basel). 2022 Nov 10;11(11):1588. doi: 10.3390/antibiotics11111588 (PMC9686928; doi:10.3390/antibiotics11111588)
Supplement: Supplementary file 1 [file antibiotics-11-01588-s001.zip › antibiotics-1983309-supplementary.pdf]

Supplementary Materials

10-mer and 9-mer WALK peptides with both antibacterial and anti-inflammatory activities

Su-Jin Kim [1,2,†], Tae-Bong Kang [1,2,†], Dong-Hyuk Kim [1,2,†], Minho Keum [1,2], Sung-Hee Lee [3], Ji-Hun Kim [3], Sang-Hyuck Lee [1,2], Jihoon Kim [1,2], Hyuk Jung Kweon [4], Jae Won Park [5], Beom Jun Kim [5] and Hyung-Sik Won [1,2,*]

[1] BK21 Project Team, Department of Applied Life Science, Graduate School, Konkuk University, Chungju, Chungbuk 27478, Republic of Korea
[2] Department of Biotechnology, Research Institute (RIBHS) and College of Biomedical and Health Science, Konkuk University, Chungju, Chungbuk 27478, Republic of Korea
[3] College of Pharmacy, Chungbuk National University, Cheongju, Chungbuk 28160, Republic of Korea
[4] Department of Family Medicine, Konkuk University School of Medicine, Chungju, Chungbuk 27478, Republic of Korea
[5] Research Institute, EYESEL Co. Ltd., Yongin, Gyeonggi 16950, Republic of Korea
* Correspondence: wonhs@kku.ac.kr; Tel.: +82-43-8403589
† These authors contributed equally to this work.

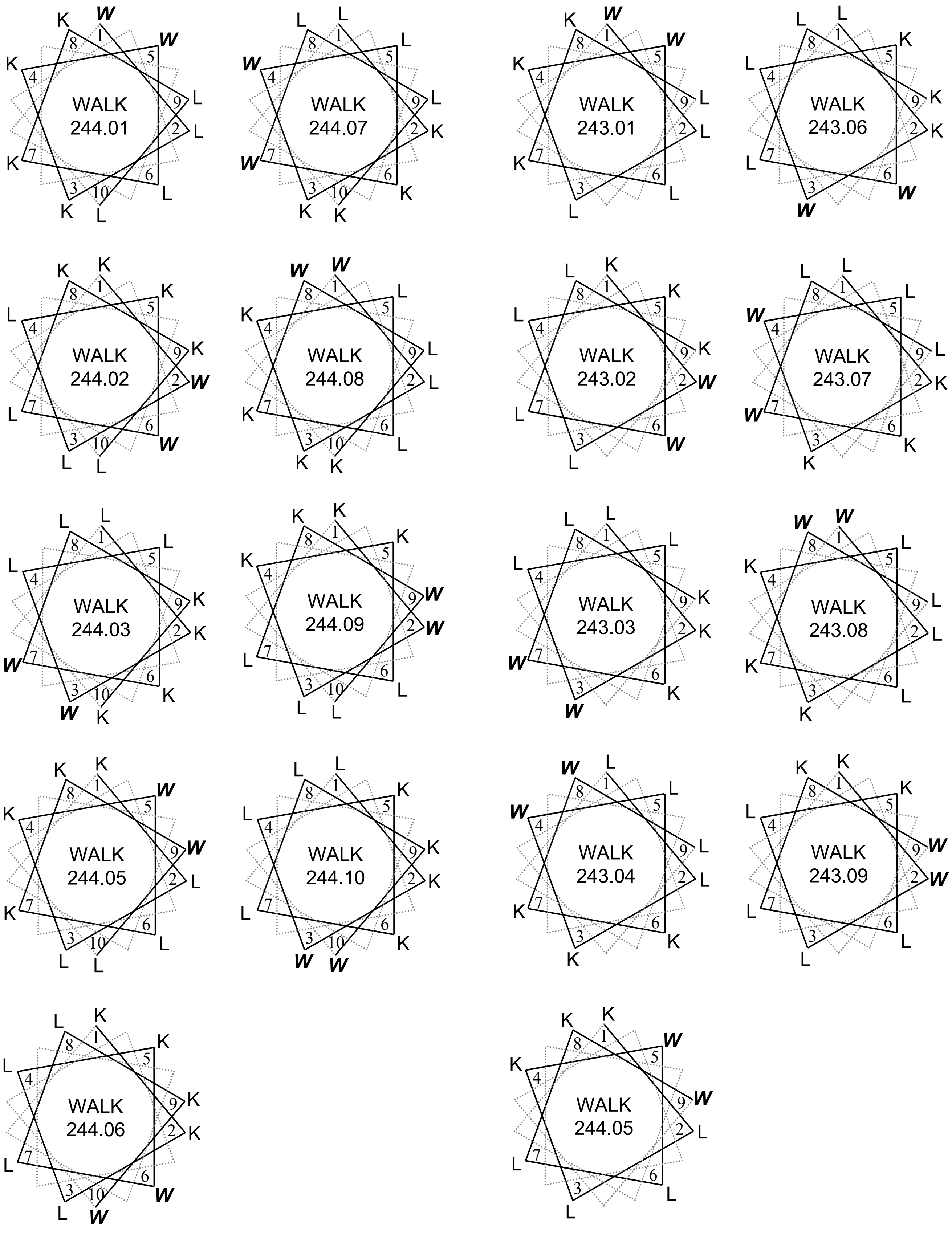

Supplementary Figure S1. Helical wheel diagrams for designing WALK244 and WALK243 peptide sequences. Tryptophan (W) residues are indicated in bold, italicized letters. The diagram for WALK244.04 is presented in the main Figure 1B.

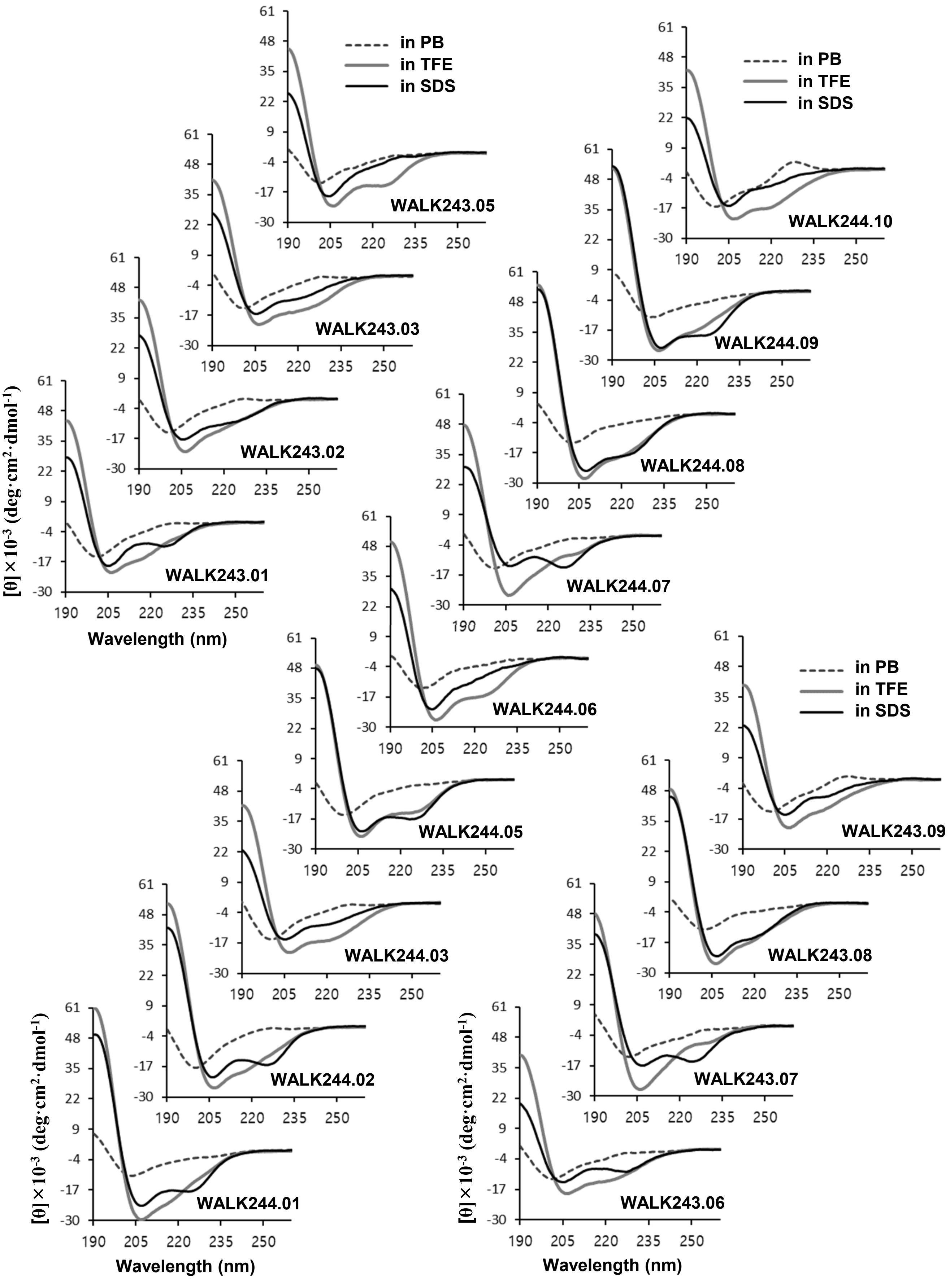

Supplementary Figure S2. Far-UV CD spectra of WALK244 and WALK243 peptides in 10 mM sodium phosphate buffer alone (dashed line; PB), PB containing 50% (v/v) trifluoroethanol (gray line; TFE), and PB containing 10 mM sodium dodecyl sulfate (black solid line; SDS). The spectra for WALK244.04 and WALK243.04 are presented in the main Figure 1C.
